# Investigation of Regression Methods for Reduction of Errors Caused by Bending of FSR-Based Pressure Sensing Systems Used for Prosthetic Applications

**DOI:** 10.3390/s19245519

**Published:** 2019-12-13

**Authors:** Chakaveh Ahmadizadeh, Carlo Menon

**Affiliations:** Menrva Research Group, Schools of Mechatronic Systems Engineering and Engineering Science, Simon Fraser University, Metro Vancouver, BC V3T 0A3, Canada; cahmadiz@sfu.ca

**Keywords:** force myography, prosthetic fitting, regression, calibration, error reduction, linear regression, random forest, general regression neural network, cross-talk, force sensitive resistor

## Abstract

The pressure map at the interface of a prosthetic socket and a residual limb contains information that can be used in various prosthetic applications including prosthetic control and prosthetic fitting. The interface pressure is often obtained using force sensitive resistors (FSRs). However, as reported by multiple studies, accuracies of the FSR-based pressure sensing systems decrease when sensors are bent to be positioned on a limb. This study proposes the use of regression-based methods for sensor calibration to address this problem. A sensor matrix was placed in a pressure chamber as the pressure was increased and decreased in a cyclic manner. Sensors’ responses were assessed when the matrix was placed on a flat surface or on one of five curved surfaces with various curvatures. Three regression algorithms, namely linear regression (LR), general regression neural network (GRNN), and random forest (RF), were assessed. GRNN was selected due to its performance. Various error compensation methods using GRNN were investigated and compared to improve instability of sensors’ responses. All methods showed improvements in results compared to the baseline. Developing a different model for each of the curvatures yielded the best results. This study proved the feasibility of using regression-based error compensation methods to improve the accuracy of mapping sensor readings to pressure values. This can improve the overall accuracy of FSR-based sensory systems used in prosthetic applications.

## 1. Introduction

The pressure profile at the interface of the prosthetic socket and the residual limb contains important information that can be used for various applications in the field of prostheses. Some of the most common prosthetic applications for which the use of this pressure map has been explored include control of powered prostheses using Force Myography (FMG) [1,2,3] and prosthetic fitting [4,5,6].

FMG for prosthetic control has been explored for both upper extremity and lower extremity prostheses [7,8]. For the upper limb, FMG has been mostly used for gesture classification to control externally-powered prosthetic hands [9,10]. The feasibility of using FMG for continuous finger movement control has also been investigated [8,11]. In lower limb prostheses, the use of FMG has been mainly focused on locomotion mode detection. Information about the mode of locomotion can be used for the ankle’s angle correction as the user walks over ramps, flat surfaces, or stairs [7,12]. Moreover, studies have shown promising results using FMG for gait phase detection [13] which can potentially be used for lower limb prostheses control [14].

Another application for which the interface pressure can be used is prosthetic fitting. Correct shaping of the socket is a critical and complex part of the prostheses design process [15]. Poor socket fit can cause various problems such as pain and discomfort [16], skin problems such as edema, dermatitis and ulcer [17] which can lead to abandonment of the prosthesis or even further surgery. These problems may also drive users to put more load on their intact limb which can cause other problems such as osteoarthritis of the knee and/or the hip joints of the intact limb [18]. Moreover, insufficient loading of the residual limb may lead to osteopenia and subsequent osteoporosis [18]. Using the aforementioned interface pressure, location of high pressure areas and the value of pressure in these areas can be determined. This information can enhance the accuracy of the prosthetic fitting process and prevent some of the problems associated with a poor socket fit.

Various force/pressure measurement techniques are used in biomedical applications. Strain gauges and load cells are used in different forms and for various applications such as measurement of ground reaction forces for gait analysis using instrumented shoes [19] and prosthetic interface pressure measurement [17]. Force plates are also commonly used in such applications due to their accuracy of measurement [19,20,21]. Other force/pressure measurement methods for biomedical applications include the use of piezoelectric sensors for measurement of normal forces in shoe insoles [19], instrumented implants for telemetric measurement of forces [22], dynamometers [23], and electromyography for measurement of muscle activation forces [21].

The measurement method is determined based on the application for which the results are to be used. For example, despite the high accuracy of measurements by a shoe insole instrumented with strain gauge transducers, it is not a suitable measurement system for gait analysis due to the interference of the thickness of the sensors with parameters of the experiment [19]. For pressure measurement within prosthetic sockets, various techniques are investigated including the use of strain gauge transducers, capacitive sensors, and piezoresistive sensors [6]. The use of strain gauge-based sensors are amongst the most accurate methods that can measure both normal and shear forces. However, factors such as the high cost of these sensors and their dimensions limit their practicality for the real use case of the applications considered in this study. The use of such sensors for prosthetic pressure measurement requires modification of the prosthetic socket to make embedment of the sensors possible. Such alterations to the prosthetic socket may affect the interface pressure distribution [24,25,26].

Capacitive sensors are used both in single point form and as sensor arrays for prosthetic pressure measurement. The rigid substrates of the single point capacitive sensors prevented them from complying with the geometry of the socket, which in addition to their costly fabrication prevented them from being an optimal technique for prosthetic pressure measurement [6]. The Pliance system by Novel Electronics (Minneapolis, MN, USA) uses capacitive sensor arrays for this application, however, the measurements are limited to direct pressure and are uni-directional [27,28].

Design and development of sensors that are thin, less costly, and can measure shear forces have been investigated in the research community. Chase et al. fabricated and tested a flexible capacitive force sensor that was able to measure normal and shear forces [29]. Razian et al. designed and developed a miniature triaxial piezoelectric copolymer film pressure transducer with thickness of 2.7 mm that can be embedded in shoe insoles [30]. Although these sensory systems can potentially be used for prosthetic pressure map registration, their development methods are still in their early stages and are bound to the research laboratories.

FSR-based pressure measurement systems are amongst the most common methods for prosthetic pressure map registration as well as a multitude of other biomedical application [31]. Despite their inability to measure shear forces which is undesirable for some prosthetic applications, their thin profile, flexibility, cost effectiveness, computationally affordable signal pre-processing [17,27,32], and commercially established development have made them a practical solution for prosthetic pressure measurement. The F-scoket system by Tekscan (Boston, MA, USA) has been one of the most commonly preferred solutions for pressure measurement inside prosthetic sockets [5,6,17,26,28,32].

Despite availability of FSR-based prosthetic pressure measurement systems in the market and their wide use in research communities, when these systems are evaluated in practical situations, higher errors are reported compared to their performance in constrained environments of the labs. In prostheses control using FMG, higher errors are reported for inter-subject and inter-trial cases [13,33]. In prosthetic fitting systems, higher errors are reported when FSR sensors are placed on moulds of the limbs [4,27,28].

Factors affecting stability of FSR responses have been investigated in multiple studies. Chegani et al. assessed the effect of sensor placement and spatial coverage on stability of FMG signals used for gesture classification through studying their effect on obtained classification accuracy. It was determined that increasing spatial coverage improved accuracy when two custom FMG bands were used instead of one. However, increasing spatial coverage beyond that did not further improve the results. They also reported that optimal placement of sensors can potentially compensate for the lower spatial coverage [8]. Delva et al. investigated the effect of anthropometry and grip strength on stability of FMG signals and determined that these factors do not contribute to variability in FMG signals. They also demonstrated that FMG signal’s stability was not decreased in non-stationary tasks [34].

Another factor that can decrease the stability of FSR signals is the curvature of sensors. In the use of FSR-based pressure sensing systems for prosthetic applications, as sensors are embedded inside a prosthetic socket, they are inevitably bent. When FSRs are bent, their neutral value changes and as the sensor responses are usually non-linear, this could considerably affect sensors’ responses.

Multiple studies have assessed the effect of bending on stability of FSR readings by studying the effect of placement of FSR sensor arrays on moulds of the limb on the sensors’ error compared with when they were laid flat [4,28]. These assessments showed that when the sensors were placed on moulds of residual limbs, their errors increased significantly compared with when they were laid flat. Three off-the-shelf FSR-based pressure sensing systems were assessed in these studies: the Rincoe SFS system, the F-Socket, and the Pliance system. Their reported accuracy error increased from 24.7% to 32.9% for the Rincoe SFS system [4], 8.5% to 11.2% for the F-Socket [4], and from 2.42% to 9.96% for the Pliance system [28].

To the best of authors’ knowledge, no study has been conducted to solve the problem of instability of FSR-based pressure sensing systems due to bending. The objective of this study is to determine feasibility of reducing errors introduced in sensor matrix readings due to the matrix being bent using information about the values of curvature of the sensors. This is a preliminary study using an off-the-shelf matrix of FSRs and five values of curvature that are uniform across the matrix. Proposed methods in this study can be applied to any system of sensor matrices that are prone to decreased accuracy when bent, including pressure measurement systems for prosthetic fitting that are available in the market. Since sensor bending affects the physical characteristic of the sensors, it could also affect other aspects of sensors’ response in addition to what was assessed in this study such as creep and hysteresis. Moreover, when sensors are placed on a residual limb, they might be bent in multiple planes. This study assessed the effect of bending in one plane. The effect of bending on creep and hysteresis in addition to multi-plane curvature of sensors should be investigated in future work.

The proposed approach for this study was to test sensor readings in a chamber with varying pressure when sensors were laid flat or when they were bent with known curvatures. Recorded data was then analyzed offline. In order to reduce the error due to bending, four regression-based error compensation methods were investigated. These error compensation methods required the use of a regression algorithm to form a model for pressure prediction based on FSR data and sensors’ curvatures. To determine which regression algorithm to use in these error compensation methods, three algorithms, namely linear regression (LR), general regression neural network (GRNN), and random forest (RF), were assessed on the data collected from the sensors in the pressure chamber. The data that were used for assessment of the three regression algorithms was the combination of sensor data in all curved conditions in addition to the flat condition. Data were split to 5 repetitions and Leave-one-out cross validation was used to chose one of the regression algorithms be used in the four regression-based error compensation methods proposed in this study. The assessment was performed based on two outcome measures: $R^{2}$ and RMSE%.

The three regression algorithms that were assessed for this study are amongst the most vastly used for regression purposes for FSR signals used in biomedical applications [8,35,36,37]. Linear machine learning algorithms are commonly used for various types of signals for a multitude of applications due to their capability of prediction and their computational efficiency [9,35,38,39]. These characteristics make LR suitable for applications that are sensitive to processing time such as the ones that require repetitions of data processing, and the applications that require real-time data processing.

GRNN is a probabilistic, memory-based neural network with a highly parallel structure. This algorithm learns in a single pass through available data and converges to the optimal regression surfaces with availability of more samples. It is capable of forming acceptable regression surfaces based on limited data and is known to work with sparse data in real-time environments. GRNN is fast learning and computationally efficient as it does not need back propagation. Moreover, its implementation is relatively simple and easy to use [36,40]. These characteristics of GRNN make it suitable for the analysis of this study, especially considering sparsity of curvature values.

RF is a non-conventional machine learning algorithm that is commonly used for classification and regression of FSR signals. It improves on individual decision trees by using an aggregation of weakly pruned trees to prevent over-fitting to training data. This enhances generalization of the models created by RF [41]. These characteristics of the three aforementioned algorithms, common application of them to FSR signals, and their performance for the baseline condition of this study, which is when data from the flat condition and various curved conditions were combined, are the reasons they were chosen for assessment of the dataset of this study.

The selected regression algorithm was used in the four regression-based error compensation methods that were investigated. The objective of the error compensation methods was to take into account the variability introduced in the sensors’ responses due to their bending. A common method to account for such variability in different conditions is to calibrate sensors separately for each condition [31]. Method1 does so by separating data based on the curvature of the sensors and making a separate model for each condition using the selected regression algorithm. To predict pressure for test data using this method, the model associated with the curvature of test data would be used. Method2 uses the selected regression algorithm to make a single model for all data. In this method, the value of curvature is used as an input channel for model training and pressure prediction. Findings of method2 motivated implementation of method3 and method4. More explanation on this is provided in the “Discussion” section. Method3 splits the data to flat and curved. It then uses the selected regression algorithm to make a separate model for each of these two conditions. Pressure prediction for test data using this method is similar to the first method. Method4 is similar to method2 except that the curvature input channel in this method has binary values of 0 and 1 representing whether the sensor is curved or flat. For comparison of performance of these methods, data were split to 5 partitions and Leave-one-out cross validation was used with the two aforementioned outcome measures ($R^{2}$ and RMSE%).

## 2. Materials and Methods

To assess the effect of bending on accuracy of the response of FSRs, sensors’ responses to known pressure values were examined as they were placed on structures that were flat or were curved with various curvatures. Regression methods were used to map sensor responses to pressure values. To assess whether bending the sensors significantly affected their response accuracy, two conditions were first compared: sensors’ responses when only the flat condition was considered and sensors’ responses when all six conditions were considered. Multiple error compensation methods were then used to decrease the errors introduced due to the bending of the sensors and their performances were compared.

To collect required data for this study, a test setup was needed that could apply pressure on sensors in the matrix while measuring the value of the applied pressure, that is, true pressure value. The measured value would then be used to produce the regression model.

For all statistical tests, normality of data was determined using the Shapiro-Wilk test. When data were from a normal distribution, depending on the number of populations being compared, either the Student’s paired *t*-test at significance level of 5% or a repeated measures ANOVA was employed. For these tests, 70 samples were used, each representing the outcome measure for one of the sensors in the matrix.

For cases where the assumption of normality was violated, non-parametric tests were used. When more than two populations were being compared, the Friedman test was used. For post hoc analysis in these cases, or in cases where two populations were being compared, depending on symmetry of the distribution of differences between paired variables, either the Wilcoxon signed-rank test or the paired-samples sign test with a Bonferroni correction was used. All tests were conducted with the IBM SPSS Statistics v24 software.

### 2.1. Sensor Matrices

**Figure 1:**
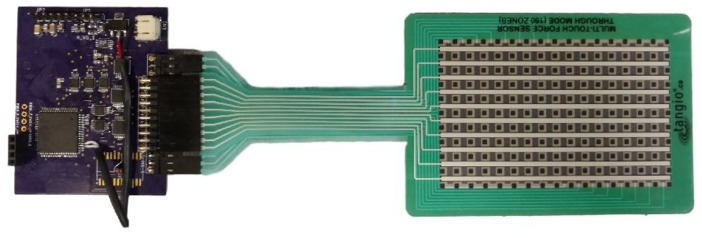
FSR matrix and the data acquisition printed circuit board (PCB) used in this study.

To explore the extent of the effect of bending on accuracy of the response of FSRs, an off-the-shelf sensor matrix of FSRs was chosen for this study: the TPE-900 Series multi-touch resistive evaluation sensor by Tangio Printed Electronics. This sensor matrix was chosen due to its independence on specific hardware or electronics. The FSR matrix used in this study was comprised of 7 rows and 10 columns of individual FSR sensors and its dimensions were approximately 7 cm × 10 cm. The sensor matrix is shown in Figure 1. For this experiment, the sensor matrix was sealed using Polydimethylsiloxane (PDMS) to prevent the pressurized air inside the chamber from filling the air channel that was integrated in the design of the matrix. If the air channel was filled with pressurized air, the pressure difference between the environment and the sensor matrix’s air channel would be zero. This would prevent the sensors from sensing the air pressure in their surrounding environment.

### 2.2. Data Acquisition

Two-dimensional (2D) networks of matrices are used in variety of applications such as tactile sensing, pressure distribution measurements, temperature sensing, gas detection, and so forth [42]. Shared signal and power lines between sensors in a row and sensors in a column allow for smaller number of traces which simplifies hardware and electronics. However, an inherent problem with the row-column fashion of these matrices is the cross-talk between adjacent elements of the matrix.

Various circuits are proposed and used in the literature for the scanning of piezoresistive sensor arrays that reduce the interference of unwanted paths [42,43,44]. Two of the commonly used cross-talk suppression circuits are based on the Voltage Feedback method and the Zero-potential method [43].

**Figure 2:**
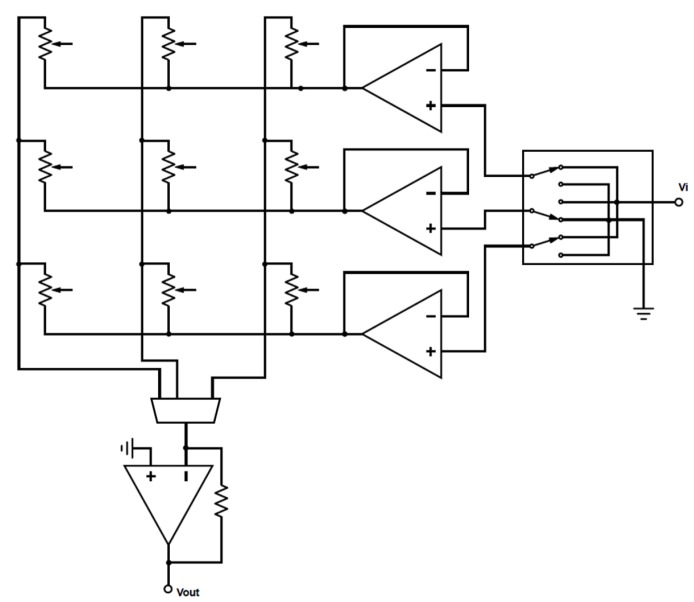
Cross talk compensation circuit used in this study.

In this study, a printed circuit board (PCB) was designed for data acquisition that used the Zero-potential circuitry shown in Figure 2. The data acquisition PCB used a Cypress PSoC 4 (model CY8C4247AXI-M485) microcontroller, op-amps, switches, a voltage regulator chip, a voltage reference chip, and multiplexers. Outputs of the circuit were transferred to a computer using universal asynchronous receiver-transmitter (UART) communication and were then saved on the computer for offline data analysis. The PCB could acquire data from sensor matrices of up to 10 columns and 16 rows. The PCB is shown in Figure 1.

True pressure values also needed to be measured and recorded. This was done using a digital pressure transducer by Omega Engineering (model PX309) whose data was acquired using a National Instrument (NI) Data Acquisition Unit (DAQ)(model USB-6001). Recordings of sensor readings and pressure sensor values were synchronized using NI LabVIEW software.

### 2.3. Test Setup

**Figure 3:**
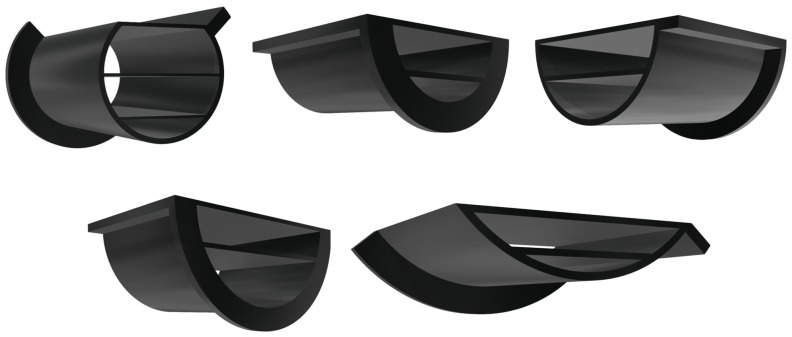
Curved structured used to bend sensor matrix with known curvatures. From left to right, top to bottom, 5, 7, 9, 11, 13 cm.

Sensors’ responses were assessed in six conditions—when sensors were laid flat and when they were placed on each of the five structures that were designed with varying curvatures. The curved structures were 3D printed and are shown in Figure 3. Each structure was part of a cylinder with a different radius, namely 5, 7, 9, 11, and 13 cm, with corresponding curvatures of approximately 0.20, 0.14, 0.11, 0.091, and 0.077 m${}^{-1}$, respectively. As transtibial amputations account for majority of the major lower limb amputations [45,46], and transhumeral amputations account for majority of the upper limb amputations [45], chosen curvatures are based on measurements of the lower transtibial residual limb reported by Persson et al. [47] and the average circumference of the arm reported by Holzbaur et al. [48].

Persson et al. studied dimensions of 93 lower residual limbs to construct a standard formula of their classification into cylindrical (ordinary), conical, and club-shaped as well as short, ordinary (breadth<length<2*breadth), or long. In this study, they tested the constructed classification formula on 96 residual limbs of 86 volunteers in 135 examinations and determined that 80% of them were ordinary in both size and shape. Measurements of the breadth of the residual limbs were not reported, however, considering the ordinary length of the majority of the residual limbs and the range reported for their length, breadth of the stump of majority of participants can be approximated to be larger than 9 cm which corresponds to the curvature of about 0.2 m${}^{-1}$ [47].

Holzbaur et al. used magnetic resonance imaging for measurement of the features of 32 upper limbs and reported an average of approximately 31 cm for the circumference of the arm which corresponds to the curvature of about 0.2 m${}^{-1}$. Based on the measurements reported by the aforementioned studies, the highest curvature used in this study was 0.2 m${}^{-1}$. The rest of the curvatures were chosen with 2 cm variations in the radius of the cylinders up to the radius of 13 cm. The reason lower curvatures were not considered is that they were not expected to have considerable effects on sensors’ responses [48]. As this was a preliminary study, it did not consider double curvatures which would be more important for the conical and club-shaped stumps which account for about 20% of the stumps according to the above study [47]. This should be considered in future work.

Design of the curved structures included fixtures to assure fixed placement of sensor matrices that was normal to the horizontal cross section of the cylinders. Fixtures were also added to the structures to ensure their mechanical stability under pressure. For the experiments in this study, to bend sensor matrices to specific, known curvatures, they were placed on these structures.

**Figure 4:**
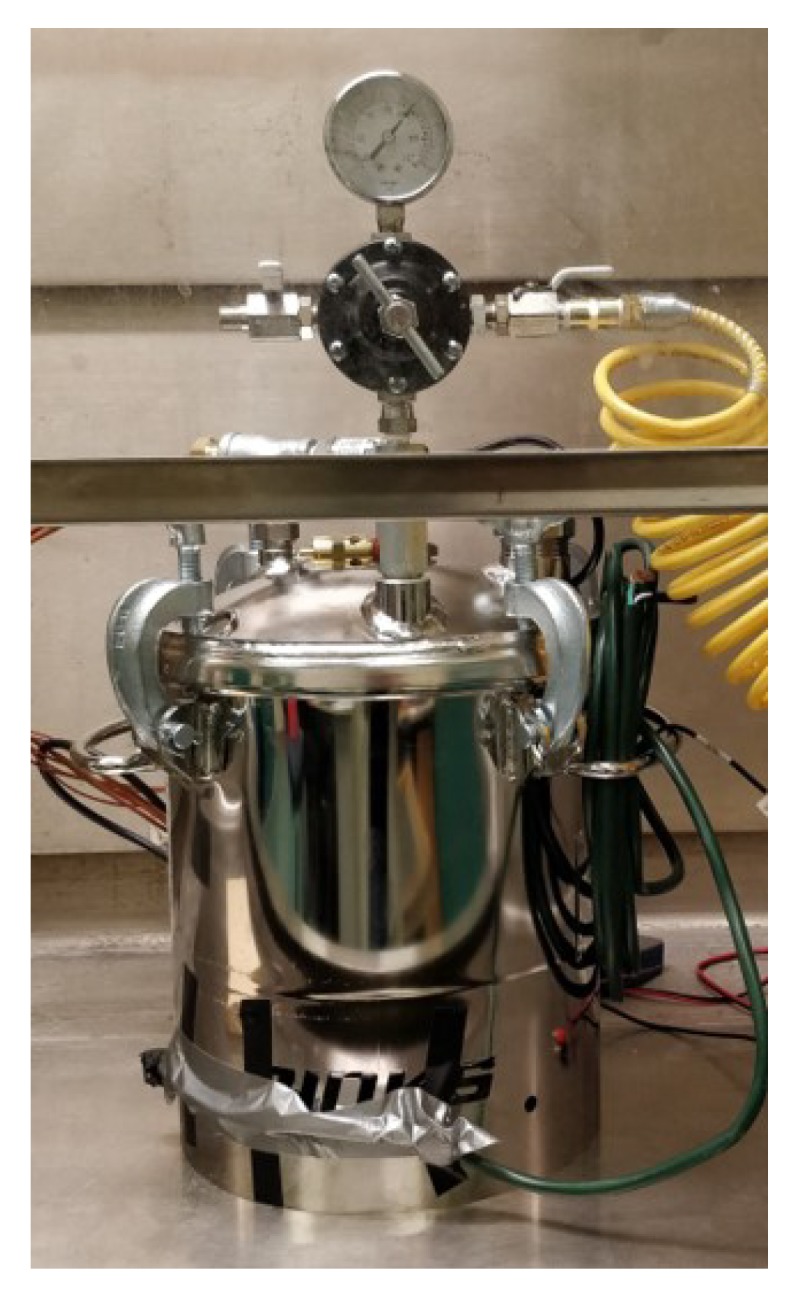
Experimental setup using a pressure chamber.

A test setup was required to apply and measure known values of pressure to the sensor matrix as it was bent. The setup included an air pressure chamber rated at 793 kPa built to American Society of Mechanical Engineers’ specifications, a pressure transducer with ±0.25% best straight-line accuracy and range of approximately −103 to 1034 kPa, and an electrical feed-through to allow for powering the system and reading output values. This setup was similar to the one used in literature to evaluate sensory systems for prosthetic fitting [4,28]. The test setup is shown in Figure 4.

### 2.4. Data Collection

The sensor matrix was placed on a flat or curved surface and was placed inside the chamber. Sensors were then tested by increasing air pressure inside the chamber up to about 620 kPa and decreasing it back to room pressure in a cyclic manner. This was repeated for each condition (five curved surfaces and the flat surface) for up to 10 cycles. This method was similar to what has been done in literature for assessment of accuracy of similar systems [4].

Frequency of data collection was 10 Hz. At each frame, both sensor values and the pressure inside the chamber were recorded. Total number of data samples (observations) for the 6 sets combined was about 45,000.

### 2.5. Regression Methods

To determine which regression algorithm to use in this study, three algorithms, GRNN, LR, and RF, were applied to collected data and their performance was compared using two outcome measures.

Linear regression uses a linear combination of input data to create the regression model as shown in the equation below [49]:

(1) $$y=\omega_{0}+\sum_{i=1}^{N}\omega_{i}x_{i},$$

where $\omega_{i}$ represents the weight of input feature *i*, $x_{i}$s are the input features, *N* is the number of features, *y* is the predicted value, and $\omega_{0}$ is the intercept of the linear model. In this study Matlab’s implementation of LR was used. No parameter turning was required.

GRNN functions based on two layers other than the input and output layers: a pattern layer and a summation layer. First, the pattern layer assesses the relationship between each input feature and the corresponding prediction value. Then, the summation layer performs a dot product of a vector containing produced signals in the previous layer and a weight vector. This layer consists of two neurons: a numerator neuron that is the summation of weighted target values; and the denominator neuron which is the summation of weight values. The mathematical representation is the following [36,40]:

(2) $$\begin{array}{cc} \; & Y\left(x\right)=\frac{\sum_{j=1}^{N}Y_{j}L\left(x,x_{j}\right)}{\sum_{j=1}^{N}L\left(x,x_{j}\right)}\\ \; & L\left(x,x_{j}\right)=exp\left[\frac{-D_{j}^{2}}{2\sigma^{2}}\right], \end{array}$$

where Y(x) is the prediction value, *x* is the new input, $x_{j}$s are the training samples, $D_{j}^{2}$ is the Euclidean distance between *x* and $x_{j}$, and σ is the spread constant that was tuned to $2^{-7}$ using a grid search. The smaller the distance between new test data *x* and the training sample $X_{j}$ is, the larger the value of $L(x,x_{j})$ becomes. This makes the effect of training samples that are more similar to the new test data greater on its predicted value.

The RF algorithm functions based on a modified version of decision trees. It creates an ensemble of weakly pruned trees. In a standard decision tree, each split is based on all available features while in RF trees, decision splits are based on comparisons of guesses among randomly selected input features. To perform prediction on a data point after the RF model is learned, the aggregation of prediction of all decision trees are used. In the implementation used in this study, the mean of prediction of trees are used as the predicted pressure [8,41]. Matlab’s implementation of RF was used in this study. A grid search was performed to tune the number of trees to 150. The default option was used for the number of features used for each decision split which is one third of the number of variables.

The two outcome measures used in this study were: coefficient of determination ($R^{2}$) and Root Mean Square Error Percentage (RMSE%) that are calculated using the following formulas:

(3) $$R^{2}=1-\frac{\sum_{k=1}^{n}{\left(y_{k}-y_{k}^{\prime }\right)}^{2}}{\sum_{k=1}^{n}{\left(y_{k}-\;\bar{\;y_{k}\;}\;\right)}^{2}}$$

(4) $$RMSE\%=\frac{\sqrt{\frac{1}{n}\sum_{k=1}^{n}{\left(y_{k}-y_{k}^{\prime }\right)}^{2}}}{range_{y}}*100,$$

where $y_{k}$ is the expected value of the reading, $y_{k}^{\prime }$ is the predicted value, $\;\bar{\;y_{k}\;}\;$ is the mean of expected values, *n* is the number of observations, and $range_{y}$ is the range of values in observations of expected values. $R^{2}$ and RMSE% are commonly used for assessment of performance of regression methods [50]. Based on these outcome measures, one of the regression algorithms was chosen to be used in this study.

Four different regression-based error compensation methods were examined to reduce the error caused in sensors’ responses due to their bending. These methods were compared with each other and the baseline results based on the two aforementioned outcome measures. Baseline values were obtained by combining data from all 6 conditions (flat and curved). In the baseline method, one regression model was created and used for the combined data. In order to eliminate any bias based on the number of samples used in different regression methods, data were down-sampled in any of the methods that were using data from multiple conditions. The four error compensation methods are described in Table 1:

In all methods data were normalized based on the mean and the standard deviation of training data before model was made. This was done since in practice, test data is unknown and cannot affect these factors. Each sensor was analyzed separately in all methods and the means of outcome measures for the 70 sensors were reported to represent the outcome measures for each method.

Leave-one-out cross validation method was used for all assessments done in this study. Data were split to 5 sections. Five repetitions of the assessment were done, in each, one of the 5 sections of data was held out as test data and the rest was used as training data. Obtained values of the outcome measures for each sensor was the average of the five repetitions.

## 3. Results

**Figure 5:**
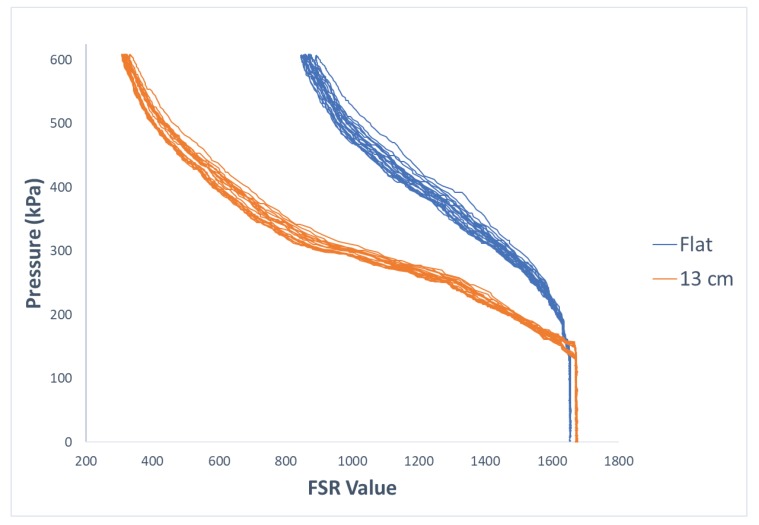
Comparison of the response of one of the FSRs when sensor was laid flat vs. when it was placed on the curved structure with radius of 13 cm.

Figure 5 illustrates how the curvature of an FSR sensor affects its response to applied force or pressure. Since such variations can negatively impact the stability of sensor readings in practical situations, we propose a regression-based calibration system that takes into account the information about the curvature of a sensor in addition to its pressure measurements. In this section, results of our proposed method are explained in detail.

### 3.1. Algorithm Selection

**Figure 6:**
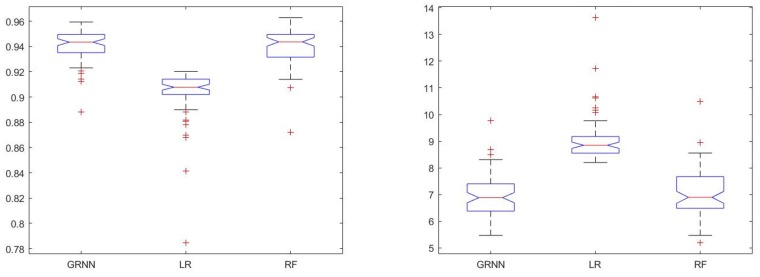
Comparison of performance of three regression algorithms: general regression neural network (GRNN), linear regression (LR), and random forest (RF). The results of the two outcome measures are shown in this figure: $R^{2}$ in the right figure and RMSE% in the left one.

Results of the two outcome measures using the three regression algorithms assessed in this study are shown in Figure 6.

Statistical analysis showed significant differences between results of the three algorithms in terms of both $R^{2}$ ($\chi^{2}\left(2\right)=107.31,p<0.001$) and RMSE% ($\chi^{2}\left(2\right)=109.83,p<0.001$). Post hoc analysis determined that LR had significantly worse performance in terms of both outcome measures. Means of $R^{2}$ values obtained using GRNN and RF were also significantly different. However, significance of the difference between GRNN’s and RF’s performances in terms of RMSE% was not determined.

GRNN was chosen as the regression algorithm to be used moving forward due to its better performance, lower standard deviations of errors and ease of use.

### 3.2. Method Selection

The comparison of when only the flat condition was considered versus when data from all six conditions were combined yielded results shown in Table 2.

**Figure 7:**
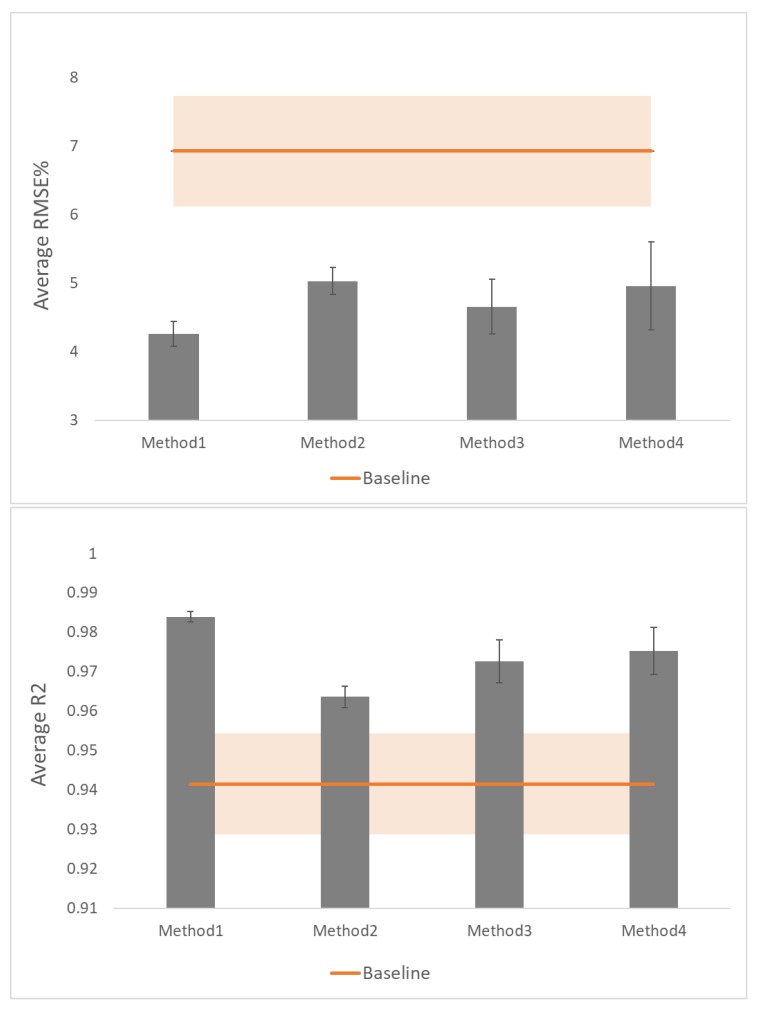
Outcome measures for the four proposed methods. The results of the two outcome measures are shown in this figure: RMSE% in the top figure and $R^{2}$ in the bottom one.

These results determined that inclusion of varying curvatures statistically reduced accuracy of prediction based on both outcome measures. To compensate for this effect, the four error compensation methods explained in Table 1 were used. Results obtained using these methods are shown in Figure 7 and Table 3.

The baseline method had the worst performance compared to all the other methods. Method1 yielded the best results. Method2 made significant improvement compared to the baseline results, however, its performance was significantly worse than method1. Method3 showed improvement over both the baseline and method2 with significant difference for both $R^{2}$ and RMSE% outcome measures. Yet it did not achieve as much improvement as method1. Method4 also showed improvement over method2 but could not reach the amount of improvement obtained using method1. Means of the average values for both outcome measures for this method were statistically significantly different from both method2 and method3. This method performed better in terms of $R^{2}$ but worse in terms of RMSE% compared with method3 with significant difference.

Statistical analysis using the Friedman test determined significant differences between results of all methods including the baseline in terms of both $R^{2}$ ($\chi^{2}\left(4\right)=270.09,p<0.001$) and RMSE% ($\chi^{2}\left(4\right)=246.83,p<0.001$). Post hoc analysis of means of both outcome measures showed significant differences between all methods.

## 4. Discussion

**Figure 8:**
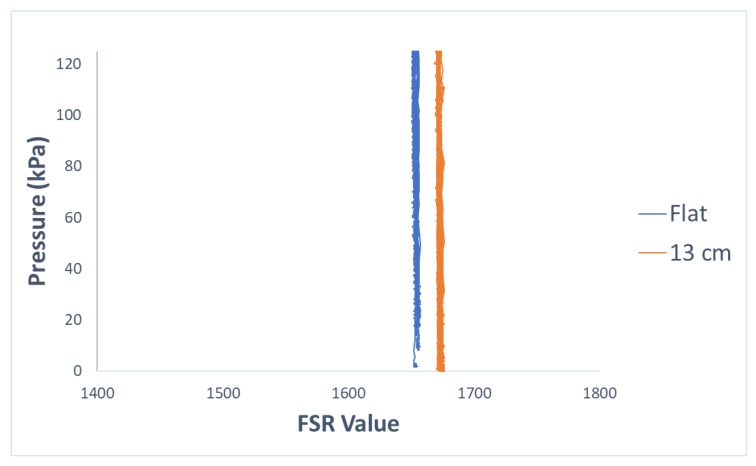
Comparison of the response of one of the FSRs when sensors were laid flat vs. when they were placed on the curved structure with radius of 13 cm. Response of the sensors in low pressure values is shown.

**Figure 9:**
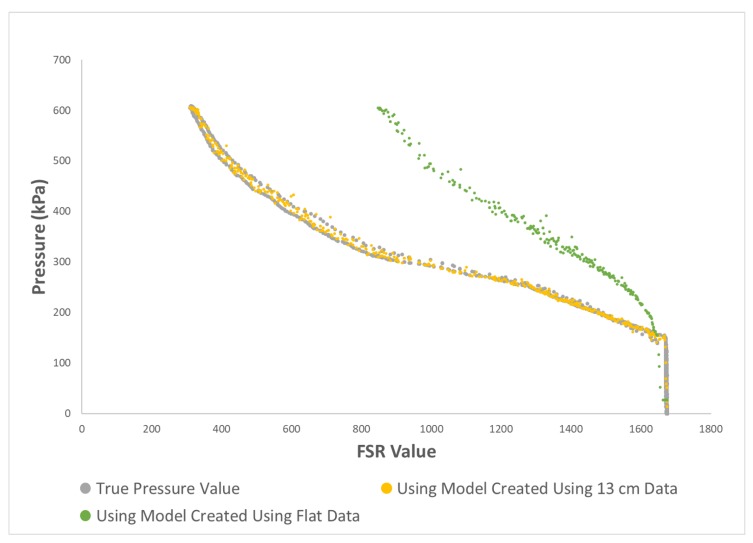
Comparison of predicting pressure from output of one of the sensors without and with considering sensor’s curvature information. Sensor was curved with a curvature of approximately 0.77 m${}^{-1}$. GRNN regression algorithm was applied to predict pressure values from the measurements of one of the sensors.

As mentioned in the ‘Introduction’ section, bending FSR sensors can affect their neutral state value which is their response in the minimum pressure of the system. The effect of bending on the neutral state value of sensors is shown in Figure 8 by comparing the response of one of the sensors in a low range of pressure when the sensor matrix was laid flat versus when it was placed on the structure with the radius of 13 cm. This phenomenon, in addition to other factors such as the non-linearity of sensors’ response to applied pressure, can considerably affect the response of FSRs when they are positioned with various curvatures. To better highlight this point, Figure 9 shows how applied pressure can be inaccurately interpreted from FSR readings if the sensor’s curvature is not taken into account. Figure 9 also shows how a regression-based calibration method can resolve this issue.

The goodness of fit of the regression models was compared in two cases—when only the flat condition was considered versus when varying curvatures were also included. This comparison determined that the effect of variation in curvatures of the sensors on their responses was statistically significant. In this step of the experiment, statistical significance was determined using the Student’s paired *t*-test.

Four regression-based error compensation methods were used to compensate for this effect. These methods were compared with the baseline. The baseline method yielded values for errors without any attempt to compensate for the bending errors in the sensor matrix. As expected, this method had the worst performance compared to all the other methods.

Method1 classified data to six classes of matrix positioning: one for flat and five for bent with different curvatures and created a separate model for each class. This method resulted in the best accuracy of prediction based on both of the outcome measures used in this study. This was likely because in this method, data from each condition was considered separately without any effect from data from other conditions. In this method, variation of data used for training and testing of each model was minimal compared to other methods. The main disadvantage of this method was its difficulty of implementation in practice. This is because, in practice, curvature values are continuous while this method requires classification of data into discrete curvature conditions. A solution for this is to classify data based on ranges of curvatures and to use separate models for each of the classes of curvature range.

**Figure 10:**
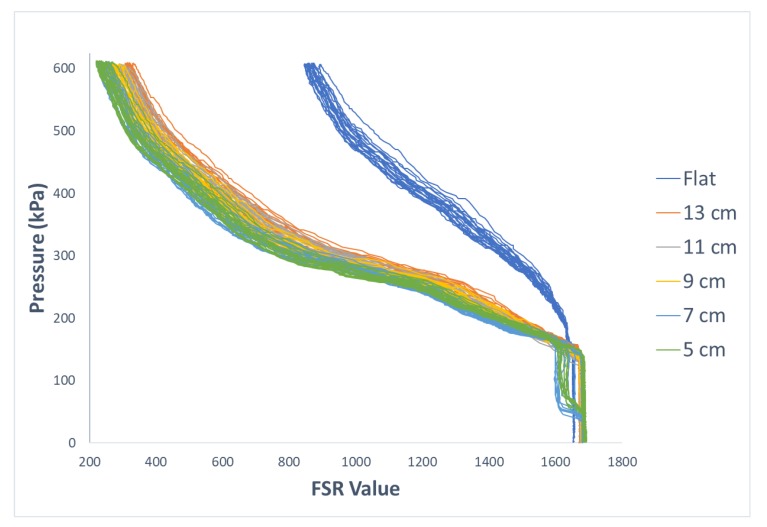
Comparison of the response of one of FSRs when sensors are positioned on structures with varying curvatures.

Method2 used values of curvatures of the sensor matrix as an added input channel to the regression algorithm. Compared to method1, this method performed significantly worse. A closer look at the data indicated that similarities between the data when sensors were curved with different curvatures was much more than similarity of the data between any of the curved conditions and the flat condition. This can be seen in Figure 10.

Looking at the physics and operation of FSRs may help in understanding why their behaviour changes when they are curved. It may also clarify the reason for similarity of sensors’ responses when curved with various curvatures. Force sensitive resistors are resistive polymer-thick-film (RPTF) sensors comprised of multiple layers including semi-conductive layers and electrode layers. These sensors often employ a spacer mechanism such as spacer layers or air channels to control the spacing between the substrates of the sensors. This layer ensures high resistance of the sensors in the absence of external forces. When force is applied to the sensors, their resistance decreases due to two main factors. The first factor is the comprising layers of the sensors becoming in contact with each other. The other factor is variation in the geometry of the semi-conductive layer in a way that reduces sensors’ resistance [35].

When sensors are bent, their physics that play an important role in their responses to pressure also change. Curving FSRs causes their comprising layers to become closer to each other which can be considered similar to pre-loading the sensors. Moreover, since the forces due to bending are not distributed evenly across the sensors [40], their curvature affects their responses not only by pre-loading the sensors, but also by changing the rate of the change of their responses to increasing pressure. It is likely that the similarity of the responses of curved sensors regardless of the value of their curvatures is because of the similarity in distribution of bending forces across sensors in these conditions compared to when they are laid flat.

The reason method2 did not make as much improvement as expected is likely that the differences between curvature values are not good representatives of the variation in data in corresponding conditions. The model likely assumes that the difference between the value of the curvatures of two conditions determines the difference between the sensors’ responses in those conditions. However, this is not the case according to the data collected for this study. For example, the difference between the curvatures of the flat condition and the curved condition with the radius of 13 cm is about 0.08 m${}^{-1}$ and the difference between the curvatures of the curved condition with the radius of 13 cm and the one with the radius of 7 cm is about 0.07 m${}^{-1}$. The differences between the value of curvatures in these cases are comparable, so the model likely assumes that the variations of the sensors’ responses in these cases would also be similar. However, looking at the graph in Figure 10, it can be seen that the sensors’ responses are much more different in the former case compared to the latter one.

A solution for this could be to use binary inputs for curvatures. This would imply using 6 extra input channels for model creation. In each of the 6 conditions, a unique binary sequence consisting of a single 1 and five 0s would be used. This solution was implemented for the dataset of this experiment. Obtained results were 0.96 ± 0.003 and 5.05% ± 0.19% for the $R^{2}$ and RMSE% respectively. Both outcome measures were improved compared to method2, however, significant improvement was not determined using the Student’s *t*-test for either of the outcome measures. To further improve on this, categorical inputs could be used for curvature. However, in that case, curvature values would need to be classified to curvature ranges which would entail similar problems as the ones explained for method1.

Another solution to improve on method2 would be to determine a mapping of curvatures to continuous values that would be able to accurately represent the extent of their effect on variation of FSR responses. This should be investigated in future work.

The similarity between all curved conditions compared to the flat condition brought up the possibility of grouping all curved data and simply separating the two situations when the sensor matrix was laid flat and when it was curved, regardless of the amount of its curvature. This led to method3 and method4. Both method3 and method4 improved on the results obtained using method2 significantly.

Method3 made two different models, one for flat and one for curved. This method yielded improvement over method2 with significant statistical difference for both R2 and RMSE% outcome measures. This is likely because the error caused by ignoring variations in data when sensors were bent with various curvatures is smaller than the error caused by assuming that the value of curvatures were accurate representatives of the amount of variation introduced in data as sensors were bent. This was expected as discussed before. Method3 did not achieve as much improvement as method1 since, in method1 the error caused by assuming no variation in data from various curvatures was also omitted.

Method3 is easier to implement in practical situations compared to both previous methods. This is because there is no need to know the exact or even approximate value of curvatures, as long as it is known that the matrix is curved. It is reasonable to assume that sensors are bent in most locations when the matrix is embedded in a prosthetic socket.

Method4 inputted an extra channel to the algorithm. The value of this channel was 0 when the sensors were laid flat, and it was 1 when they were curved regardless of the amount of bend. This method, similar to method3 groups all curved data together. Method4 also showed improvement over method2 but could not reach the amount of improvement obtained using method1 for the same reasons explained for method3.

It is worth noting that, since only two values, 0 and 1, were used as the second input for method4, this method is similar to binary curvature inputs that was discussed for method2. This is likely the reason why method3 and method4 have comparable performances (each outperforms the other based on one of the outcome measures). Method3 performed significantly better than method4 in terms of RMSE%, however, it performed significantly worse than method4 in terms of $R^{2}$. In comparison of the last two methods, that is, method3 and method4, it was determined that method3 outperformed method4 based in RMSE% while method4 outperformed method3 based on the other outcome measure ($R^{2}$). Because we only considered two outcome measures in this study, neither of which was considered more important than the other, and due to the fact that each of these methods performed better that the other based on one of these outcome measures, we cannot conclude superiority of one of these methods compared with the other. As a result, we cannot consider one of them to have had better overall performance for the dataset of this study.

In terms of computing power and running time, variations for testing using these methods are not considerable. This is because in all methods, the model is produced using offline data. In online testing, at each sample and for each sensor, one prediction is performed using the pre-built model. The main difference among different methods would be in the training time. However, this is not an important factor in applications considered in this study since model production would be performed offline.

In order to use the proposed methods of this study, information about curvature of the limb is required. Various methods are used for geometric assessments of a residual limb that can be used for this purpose. Some possible methods include circumferential measurements and contacting methods utilizing digitizing methods of the cast of the limb or the residual limb itself [51,52]. Since, in common practice for the fabrication of prosthetic sockets, casts are made in one of the initial steps, cast of the limb is available and can be used for geometric assessment of the limb [53]. Three-dimensional (3D) scanning is another method that has recently gained attention in the field of prosthesis for various applications. 3D scans of the limb can be used for extraction of information required for the methods proposed in this study. Another option would be to place sensors on the cast of the limb and calibrate them as they are curved and placed on the location of the limb that they would later be positioned on for measurements.

Bend sensors can also potentially be used to measure the value of curvature of sensors. Compared to the aforementioned methods, the use of bend sensors can be faster and easier but less accurate. Another method that could be valuable for this purpose in the settings similar to the one used in this study, is to use the value of FSRs in specific states to determine their curvature value. To achieve this, values of sensors in room pressure or another known state could be considered. Another option would be to determine the curvature value based on the response of sensors to cyclic variations of pressure. In either of these cases, features should be extracted from time windows with optimized lengths. For more reliable prediction of curvatures, values of multiple adjacent sensors can be considered. This method should be investigated in future work.

## 5. Conclusions

In this study, it was determined that when FSR sensors were bent, the error in their mapping to pressure values significantly increased compared to when they were laid flat.

Four regression-based error compensation methods were proposed to solve the problem of instability of FSR sensor matrices when placed on a curved structure. All proposed methods significantly improved the accuracy of mapping of sensor readings to pressure values. It was determined that by just knowing when the sensor was curved without any knowledge about the value of its curvature, results could be improved using proposed methods in this study.

The best performance was achieved by making separate models based on the value of curvature of sensors. The second best performance was obtained by grouping all curved conditions and separating them from the flat condition. The former method would require classifying curvature values to pre-defined ranges. This is to be investigated in future work. The latter method is simpler and more practical since no knowledge about the value of the curvature is required, however, the amount of improvement obtained using this method is significantly less than the former method.

Based on findings of this study, for practical use of the proposed methods, method1 is recommended due to its better performance in terms of both outcome measures. To use this method with continuous curvature values, curvatures would be grouped into classes of different ranges and a model would be made for each group. Then, depending on the range that the sensor’s curvature falls into when placed on the limb, the regression model for its calibration would be selected. More investigation is required to determine the optimum ranges of curvatures.

## 6. Limitations and Future Work

This was a preliminary study that demonstrated feasibility of using the proposed methods to increase robustness of FSR-based systems for prosthetic applications. Some of the limitations of this study and future work are explained in this section.

Experimental conditions such as sensor sealing with PDMS may have affected the reported results. This reduces generality of the findings of this study. Various types of FSR sensors, for example sensors that do not need to be sealed, should be tested in different experimental conditions to confirm findings of this study in future work.

In order to fully control the experiments carried out in this study, the sensors were not removed in between tests. Next steps in future work, should assess the effect of removing and re-positioning sensors between multiple repetitions of data collection. This would be to assess the effect of variability of data due to re-positioning on accuracy of pressure prediction using the methods proposed in this study. The use of proposed methods for sensors incorporated into prosthetic systems should also be investigated and clinical tests of these systems should be conducted.

In future studies, more curvature values need to be tested to determine whether even with very low values of curvature, these methods would prove useful. Continuous curvatures need to be examined to obtain a more generalized dataset. Moreover, more complex shapes need to be tested in future work that would cause different sensors in a matrix to have different amounts of bend. This would be to mimic the scenario when sensor matrices are placed on a residual limb. An experiment should be conducted in which sensors are placed on a mould of the residual limb inside the pressure chamber and models are built according to data recorded in this situation. Moreover, these methods should be examined using a variety of sensor matrices and single FSRs.

To further build on findings of this study, the effect of hysteresis and creep on FSRs should also be investigated. Compensation methods should be proposed to enhance stability of these sensors in practical and prolonged prosthetic applications.

## Figures and Tables

**Table 1:** Regression-based error compensation methods used to reduce the errors introduced due to bending of the sensors.

**Method1**	A separate model was made for each curvature and data for each curve was assessed separately. A total of 6 models were used in this method.
**Method2**	The value of curvature for each of the curved or flat structures was inputted as an extra channel to the model. Data was down sampled to 1/6 of total observations so that the number of observations was comparable to the one for Method1.
**Method3**	All curved structures were grouped together, and two models were made in total: one for when the matrix was laid flat, and one for all the curved conditions. Data was down sampled so that the total amount of data for each of the two groups was comparable to the one in method1.
**Method4**	Similar to method2, an input was added for curvature values. However, value of curvature was set to 0 for flat, and 1 for all the curved conditions. Data was down sampled similar to method3.

**Table 2:** Comparison of when sensors were laid flat vs. when all 6 conditions were considered.

Method	$R^{2}$	RMSE%
**Flat**	0.99 ± 0.0012	3.51 ± 0.19
**All Curvatures**	0.94 ± 0.013	6.93 ± 0.81

**Table 3:** Results of method selection.

Method	$R^{2}$	RMSE%
**Baseline**	0.94 ± 0.013	6.93 ± 0.81
**Method1**	0.98 ± 0.0014	4.26 ± 0.18
**Method2**	0.96 ± 0.0027	5.03 ± 0.20
**Method3**	0.97 ± 0.0055	4.66 ± 0.40
**Method4**	0.98 ± 0.0060	4.96 ± 0.64

## Abbreviations

The following abbreviations are used in this manuscript:

FSR	Force Sensitive Resistor
FMG	Force Myography
PDMS	Polydimethylsiloxane
GRNN	General Regression neural network
LR	Linear Regression
RF	Random Forest
RMSE	Root Mean Square Error
PCB	Printed Circuit Board
UART	Universal Asynchronous Receiver-transmitter
